# Coelimycin inside out — negative feedback regulation by its intracellular precursors

**DOI:** 10.1007/s00253-024-13366-1

**Published:** 2024-12-10

**Authors:** Magdalena Kotowska, Mateusz Wenecki, Bartosz Bednarz, Jarosław Ciekot, Wojciech Pasławski, Tomasz Buhl, Krzysztof J. Pawlik

**Affiliations:** 1 Hirszfeld Institute of Immunology and Experimental Therapy, Polish Academy of Sciences, Rudolfa Weigla 12, 53-114, Wroclaw, Poland; 2 Faculty of Biotechnology, Laboratory of Biological Chemistry, University of Wroclaw, Fryderyka Joliot-Curie 14a, 50-383 Wroclaw, Poland; 3 Laboratory of Translational Neuropharmacology, Department of Clinical Neuroscience, Karolinska Institutet, Stockholm, Sweden

**Keywords:** Major facilitator superfamily, *Streptomyces coelicolor*, Secondary metabolism, Polyketide, Regulation, Reporter system

## Abstract

**Abstract:**

Coelimycin (CPK) producer *Streptomyces coelicolor* A3(2) is a well-established model for the genetic studies of bacteria from the genus *Streptomyces*, renowned for their ability to produce a plethora of antibiotics and other secondary metabolites. Expression regulation of natural product biosynthetic gene clusters (BGCs) is highly complex, involving not only regulatory proteins, like transcription factors, but also the products of the biosynthetic pathway that may act as ligands for some regulators and modulate their activity. Here, we present the evidence that intracellular CPK precursor(s) (preCPK) is involved in a negative feedback loop repressing the CPK BGC. Moreover, we provide a characterization of the cluster-encoded efflux pump CpkF. We show that CpkF is essential for the extracellular CPK production. In order to track down which CPK compounds — intra- or extracellular — are the ones responsible for the feedback signal, a luciferase-based reporter system was applied to compare the activity of 13 CPK gene promoters in the wild-type (WT) and two mutated strains. The first strain, lacking the CPK-specific exporter CpkF (*ΔcpkF*), was unable to produce the extracellular CPK. The second one did not produce any CPK at all, due to the disruption of the CpkC polyketide synthase subunit (*ΔcpkC*). All tested promoters were strongly upregulated in *ΔcpkC* strain, while in the *ΔcpkF* strain, promoter activity resembled the one of WT. These results lead to the conclusion that the CPK polyketide acts as a silencer of its own production. Supposedly this function is exerted via binding of the preCPK by an unidentified regulatory protein.

**Key points:**

•*Intracellular coelimycin precursor takes part in a negative cpk cluster regulation*

•*CpkF exporter is essential for the extracellular coelimycin production*

•*Simple method for the analysis of coelimycin P2 production in agar medium*

**Graphical abstract:**

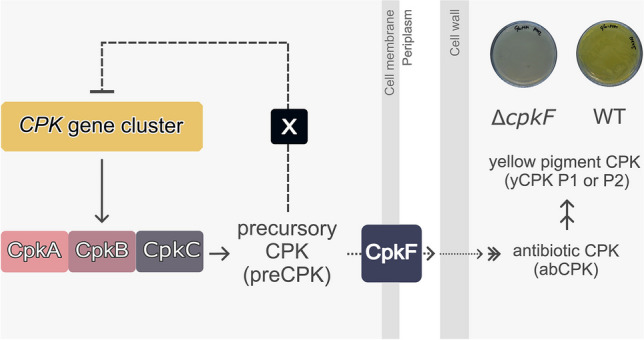

**Supplementary Information:**

The online version contains supplementary material available at 10.1007/s00253-024-13366-1.

## Introduction

Bacteria from the genus *Streptomyces* remain undefeated leaders in their ability to produce antibiotics and other secondary metabolites (SM) of great importance to human and veterinary medicine. The majority of the SM biosynthetic gene clusters (BGCs) remain “silent” until the specific signals associated with the environmental conditions and the colony development state trigger their expression. Coelimycin (CPK) cluster of *Streptomyces coelicolor* A3(2) is a perfect example of such a “silent” BGC. The expression of CPK BGC is tightly controlled by a number of pleiotropic and cluster-situated regulatory proteins (Pawlik et al. 2010; Bednarz et al. 2019). It is switched on by a quorum sensing signalling via γ-butyrolactones (GBLs), which releases the ScbR repressor from the DNA and allows for the expression of CpkO and CpkN — cluster-situated activators. ScbR2 repressor, activated by CpkO, turns off the expression of the cluster.

SMs and their biosynthetic intermediates, for example auricin (Kutas et al. 2013), sansanmycin (Li et al. 2013) and lidamycin (Li et al. 2015), can act as ligands controlling the expression of their cognate BGCs. Results of our previous work suggested the existence of a negative regulatory feedback loop involving the polyketide product of the CPK BGC (Bednarz et al. 2021). In the current work, we provide the evidence of the existence of such a feedback loop and show that the intracellular CPK precursor(s) participate in this mechanism.

**Fig. 1:**
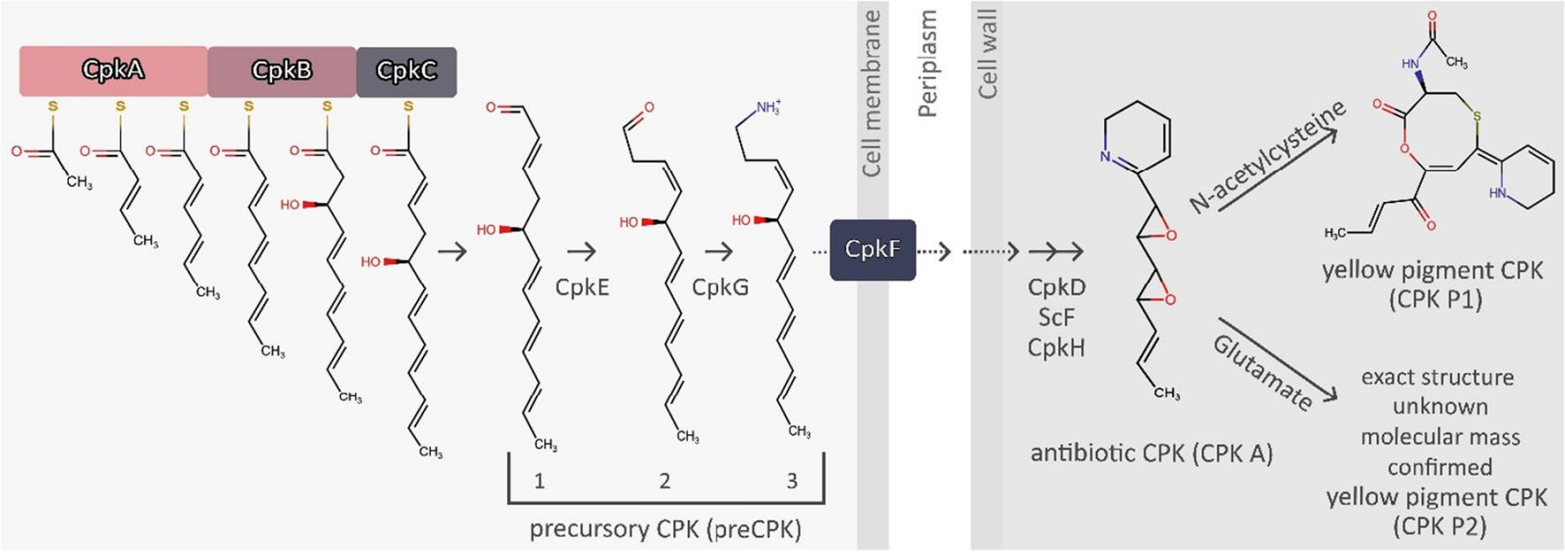
Biosynthesis of coelimycin

The scheme of CPK biosynthesis is shown in Fig. 1. The unsaturated 12-carbon chain synthesized from six malonyl residues by the type one modular polyketide synthase Cpk, composed of three multi-enzymatic subunits CpkA/B/C, is released from the megasynthase in the form of an aldehyde by the C-terminal thioester reductase domain of the CpkC subunit (Fig. 1, compound 1). It is converted into an amine derivative (Fig. 1, compound 3) by the ω-transaminase CpkG (Gomez-Escribano et al. 2012; Awodi et al. 2016). We propose the term preCPK for the 12-carbon intracellular coelimycin precursors. Further post-polyketide modifications are performed by the extracellular enzymes CpkD, ScF and CpkH. They include the formation of two epoxide rings and give rise to the compound with weak antibacterial activity (antibiotic CPK, abCPK) (Gottelt et al. 2010). Nonenzymatic reactions of the *bis*-epoxide coelimycin A (CPK A) with N-acetylcysteine or glutamate present in the medium result in the formation of coelimycins P1 or P2, respectively, which are both yellow pigments (yCPK) (Gomez-Escribano et al. 2012).

The CPK biosynthetic gene cluster from *S. coelicolor* A3(2) includes the *cpkF* gene encoding a protein with high homology to multidrug efflux pumps from the major facilitator superfamily (MFS) (Pawlik et al. 2007). The MFS is the largest superfamily of transporters, consisting of more than 100 distinct families. It was recently expanded to include five more distantly related families that do not have known transport functions (Wang et al. 2020). MFS transporters use chemiosmotic energy, often from the transmembrane electrochemical proton gradient. They operate by the mechanism of symport, where two or more solutes (one of them being H^+^ or Na^+^) are transported in the same direction: antiport, where two or more solutes are transported in opposite directions; or uniport, where a single solute is transported. MFS members have 12 or 14 transmembrane α-helical segments (TMSs), with an overall pseudo-twofold symmetry of two distinct six-transmembrane helix bundles and a substrate accommodating cavity between them (Wang et al. 2020; Drew et al. 2021). Conserved sequence motifs were identified in MFS proteins (Paulsen et al. 1996b). Some of them are shared by all MFS members, while others are family specific. Interestingly, there is no clear motif distinguishing between substrate-specific and multidrug transporters. A number of studies have investigated the roles of single amino acids in substrate recognition and transport across membranes (reviewed in Kumar et al. (2020)). Another recent extensive review (Drew et al. 2021) provided a detailed mechanistic explanation of the alternating-access mechanisms of transport, based on the available MFS structures.

Genomic analyses provide interesting data on the abundance of different transporter classes in industrially important bacteria (Lorca et al. 2007; Getsin et al. 2013; Zhou et al. 2016). As shown by a comparative analysis of 11 *Steptomyces* genomes, transporter genes constitute 10–13% of all ORFs (990 in the case of *S. coelicolor* A3(2)), and approximately one-third of them belong to the MFS (Zhou et al. 2016). Membrane transporters are often encoded within biosynthetic gene clusters, where they participate in the final steps of natural product synthesis and protect host cells producing toxic compounds (Severi and Thomas 2019). BGCs usually include at least one predicted transmembrane efflux protein. Transporter-encoding genes from biosynthetic gene clusters are often similar to those conferring antibiotic resistance in pathogenic bacteria. Transporter engineering has the potential to increase specialized metabolite production and is particularly important in hybrid biosynthetic systems construction (Severi and Thomas 2019). Despite their abundance, experimental data on the specific roles of secondary metabolite transporters are limited.

The first part of the present study focuses on the characterization of CpkF transporter from the coelimycin BGC. In the second part, addressing the feedback mechanism, CpkF (transporter)- and CpkC (main polyketide synthase subunit)-deficient strains are used as tools for tracking down the coelimycin-dependent transcriptional changes in the CPK biosynthetic cluster.

## Materials and methods

### DNA manipulation and bacterial strain growth conditions

Standard protocols were used for DNA manipulations (Sambrook and Russell 2001). Fragments amplified by PCR were first cloned into the p-GEM-T Easy vector (Promega), verified by DNA sequencing and cloned into appropriate plasmids. The oligonucleotides, plasmids and bacterial strains used in this work are listed in Tables S1, S2 and S3 in the supplementary material, respectively. The culture conditions, transformation and conjugation methods followed the general procedures for *E. coli* (Sambrook and Russell 2001) and *Streptomyces* (Kieser et al. 2000). *S. coelicolor* was cultivated on the following media: 79NG (Bednarz et al. 2021), Glu-MM (Kotowska et al. 2014) and MS (Kieser et al. 2000).

### Construction of cpkF deletion and complementation strains

The *cpkF* gene (*SCO6278*) was replaced on the St1G7 cosmid with a hygromycin resistance cassette by PCR targeting (Gust et al. 2004). The modified cosmid St1G7-cpkF_DM_ was then used for homologous recombination with the *S. coelicolor* M145 strain chromosome. The *cpkF* deletion mutant strain was named P112. The plasmids pWP4 and pIJ10257apra-cpkF were introduced into the P112 strain via conjugation with the *E. coli* ET12567/pUZ8002 strain to generate the complemented strains P127 and P321, respectively. The empty plasmids pWP3 and pIJ10257apra were introduced into the P112 strain to generate the control strains P126 and P322, respectively.

### HPLC analysis of coelimycin production

Petri plates containing 20 ml of Glu-MM solid medium were inoculated with approximately 10^8^ spores of *S. coelicolor* strains spread evenly on the agar surface. The plates were incubated at 30 °C for 27 h. The whole agar with biomass was frozen overnight at − 20 °C, thawed and centrifuged (10 min, 27,000 × g, 4 °C). The cleared supernatant was collected for HPLC analysis. HPLC analysis was performed as described previously (Kotowska et al. 2014).

### Reporter system assay

A set of promoter regions of genes from the *cpk* biosynthetic cluster was amplified from pFLUXH derivatives (Bednarz et al. 2021) with the primers GFLUX-F and GFLUX-R and cloned into the pFLUX reporter plasmid linearized with *Hind*III and *Nde*I via Gibson assembly. The promoter region of the *cpkK* gene was amplified with the primers ksTF and p6284_Nde, ligated with pUC18 digested with *Sma*I and recloned into pFLUXH digested with *Nde*I and *Bam*HI. Amplification with GFLUX-F and GFLUX-R primer pair and Gibson assembly was used to clone the pcpkK fragment into the pFLUX. The promoter regions of the *cpkI*, *cpkJ* and *accA1* genes were amplified with respective primer pairs (Table S1) from the genomic DNA of *S. coelicolor* A3(2) wild-type strain M145 and cloned by Gibson assembly into either pFLUX or pFLUXH. pFLUX derivatives with promoter regions (Table S2) were individually introduced into the M145 and P112 strains by conjugation. pFLUXH derivatives were introduced into the P100 strain. Luciferase activity was monitored for 72 h in cultures grown on solid 79NG medium in a ClarioStar Plus microplate reader (BMG Labtech) as described previously (Bednarz et al. 2021).

### Antimicrobial activity test

The antimicrobial activity of mono- and divalent cationic lipophilic compounds against *S. coelicolor* A3(2) strains was tested by the disc diffusion method. Two hundred microlitres of spore suspension in water (OD600 = 0.3) was spread on solid 79NG medium. Ten microlitres of water solutions of acriflavine hydrochloride (5 mg/ml), benzalkonium chloride (5 mg/ml), chlorhexidine diacetate (0.5 mg/ml) and pentamidine isethionate salt (5 mg/ml) was applied to Whatman GF/F sterile discs with a diameter of 6 mm. The discs were then placed on the surface of the inoculated plates. The inhibition zone diameter was measured after 48 h of incubation at 30 °C.

### Bioinformatic tools

An amino acid sequence similarity search was performed with BLAST (Altschul et al. 1990). A phylogenetic tree of CpkF and its homologues was generated with Clustal Omega (Sievers et al. 2011) and visualized with iTOL (Letunic and Bork 2021). Consensus prediction of the CpkF topology was performed by TOPCONS (Tsirigos et al. 2015). The presence of signal peptides in protein sequences was analysed with SignalP 5.0 (Almagro Armenteros et al. 2019).

## Results

### The CpkF transporter is necessary for efficient yCPK production

According to the biosynthetic pathway proposed by Gomez-Escribano et al. (2012), the precursor of CPK, (3Z,5S,6E,8E,10E)−1-aminododeca-3,6,8,10-tetraen-5-ol (Fig. 1, compound 3), in the form of a monovalent cation, is the predicted native substrate transported by CpkF. Further biosynthetic steps performed by extracellular enzymes lead to the formation of the colorless *bis*-epoxide CPK A, which can then form adducts with culture medium components. The use of the minimal medium containing glutamate (Glu-MM) allows the conversion of virtually all the precursor to CPK P2 and allows for convenient detection of the product by HPLC (Kotowska et al. 2014).

**Fig. 2:**
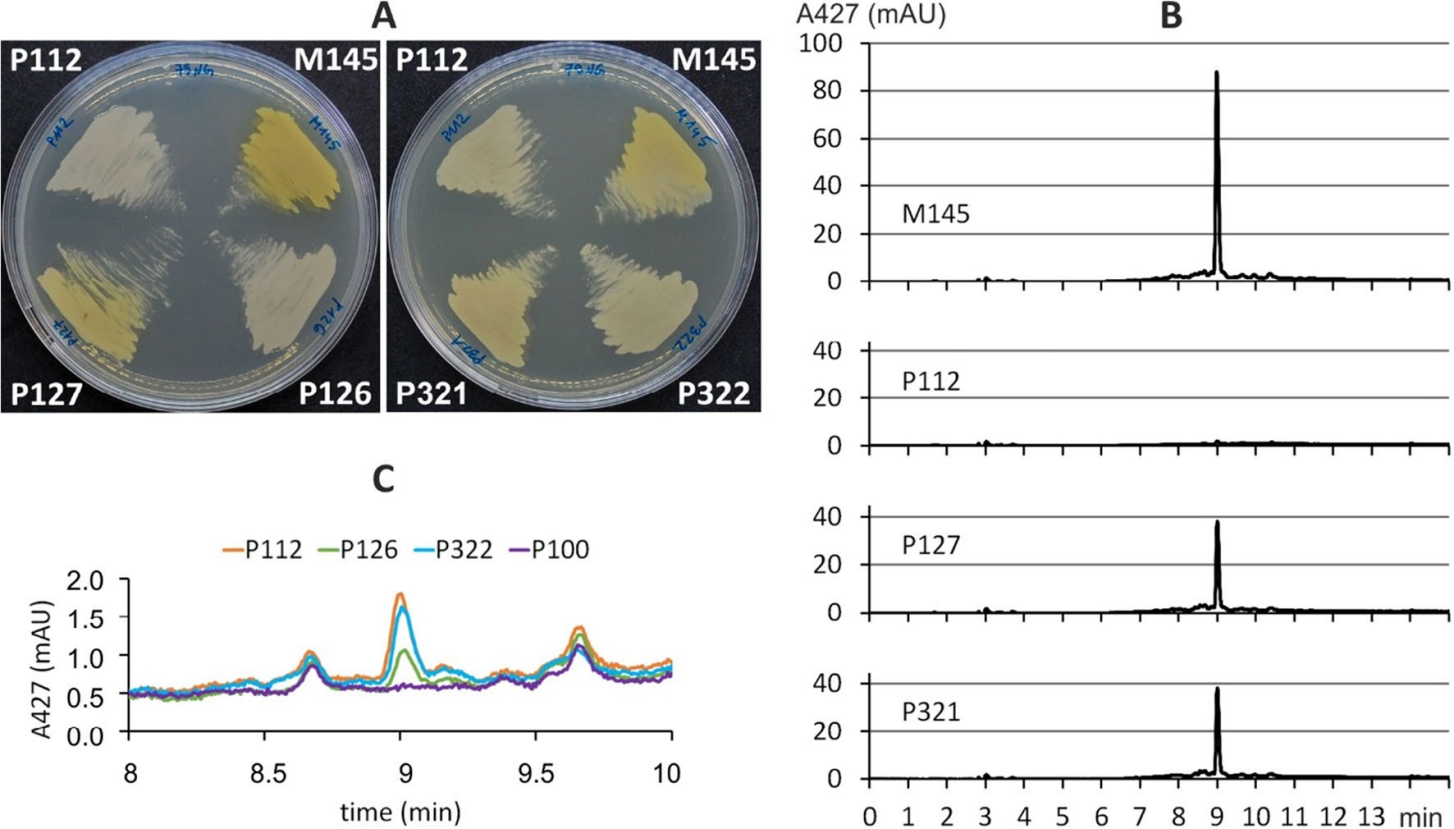
The production of coelimycin by *S. coelicolor* A3(2) strains. The strain names are the same as in Table 1. (**A**) Cultures on 79NG medium incubated for 24 h. CPK is visible as a yellow pigment. (**B**) Representative HPLC chromatograms monitoring the absorbance at 427 nm from 27-h cultures on solid Glu-MM medium. The 9-min peaks from the M145, P127 and P321 samples were confirmed by mass spectrometry as coelimycin P2. (**C**) Close-up of the residual CPK P2 peaks in the HPLC chromatograms

To investigate the role of the CpkF transporter in the CPK production, the *cpkF* gene was replaced with a hygromycin resistance cassette, yielding the *cpkF* deletion strain P112. A lack of the transporter CpkF abolished yellow CPK production. Complementation of the deletion by the *cpkF* gene under the constitutive promoter ermEp* either on an integrating plasmid (strain P321) or on a high copy number plasmid (strain P127) restored yCPK production (Fig. 2A). This was confirmed by HPLC analysis (Fig. 2B, Table 1). A single peak of A_427_, which eluted at 9 min, was observed. The 9-min peaks from the M145, P127 and P321 samples were confirmed by mass spectrometry as CPK P2.

**Table 1:** The production of coelimycin P2 by *S. coelicolor* A3(2) strains after 27 h of culture on solid Glu-MM medium measured as the area under the 9-min peak. The data are shown as the means from three plates ± standard deviation. Figure 2 shows the chromatograms

Strain	Strain description	Peak area (mAU*min)	% of wild type
M145	Wild type	7.26 ± 1.06	100
P112	*ΔcpkF*	0.09 ± 0.01	1.3
P127	*ΔcpkF* + pWP4 (complemented)	3.39 ± 1.34	46.6
P126	*ΔcpkF* + empty pWP3	0.03 ± 0.01	0.4
P321	*ΔcpkF* with pIJ10257apra-cpkF (complemented)	3.72 ± 0.53	51.3
P322	*ΔcpkF* + empty pIJ10257apra	0.13 ± 0.05	1.9
P100	*ΔcpkC*	0.0	0.0

Careful investigation of the HPLC plots revealed a low level of residual CPK P2, below 2% of the wild-type level, in the deletion strain P112 and its derivatives with empty plasmids (strains P126 and P322), as opposed to the P100 strain, which is unable to complete the synthesis of the polyketide backbone of the CPK precursor due to the knockout of *cpkC* gene encoding one of the main polyketide synthase subunits (Fig. 2C, Table 1).

### CpkF is a cluster-specific efflux pump dedicated to coelimycin

CpkF, a 543 aa protein encoded by the *SCO6278* gene from the CPK BGC of *S. coelicolor* A3(2), belongs to the major facilitator superfamily of membrane transport proteins according to its predicted amino acid sequence similarity.

**Fig. 3:**
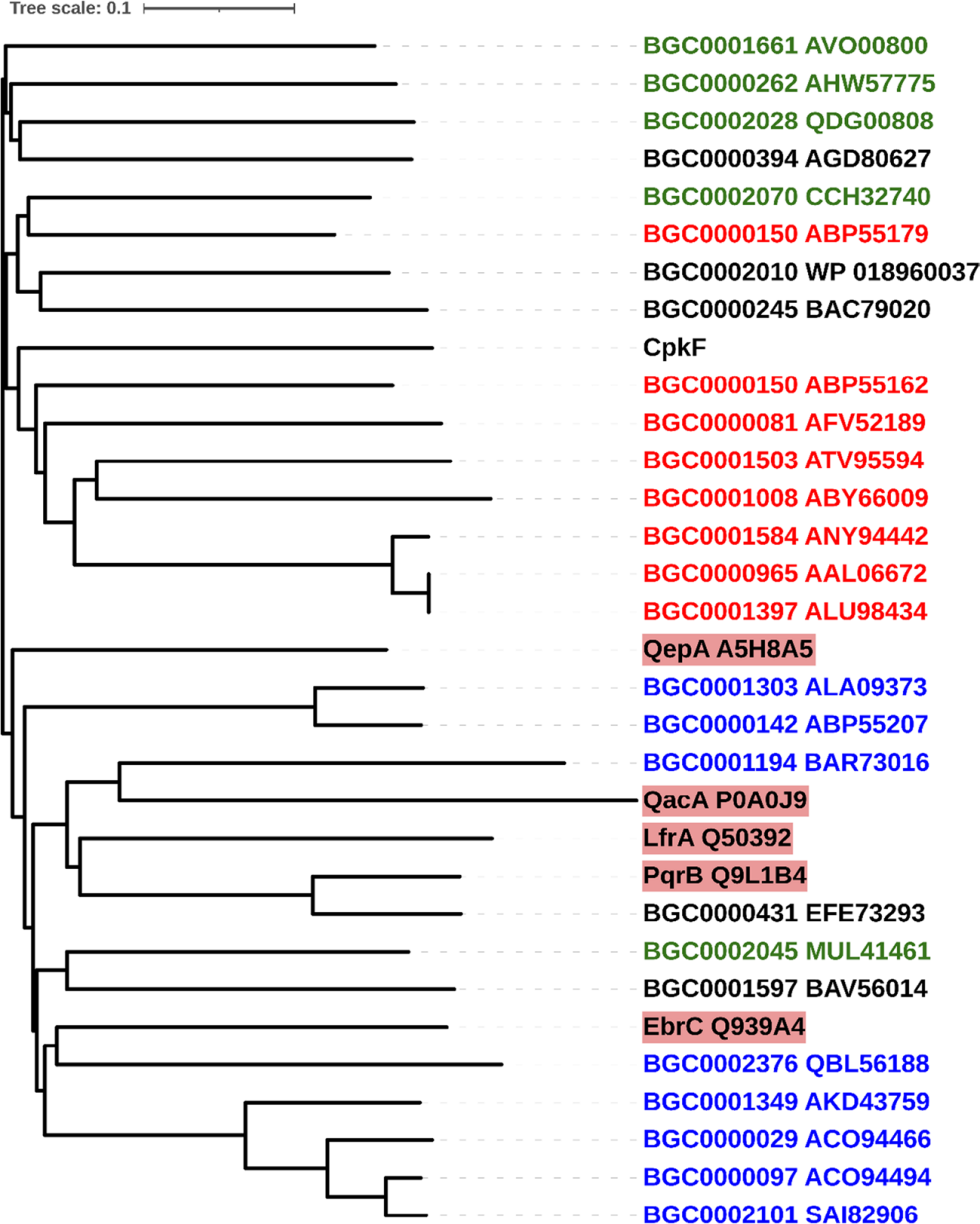
A phylogenetic tree of CpkF and its homologues listed in Tables S4 and S5 generated with Clustal Omega (Sievers et al. 2011) and visualized with iTOL (Letunic and Bork 2021). The color labels denote the classes of the main compounds produced by the respective BGCs: red, enediyne; green, angucycline and anthracycline; blue, polyene macrolactam; black, other. MFS pumps not associated with BGCs are marked with a pink background

A BLAST search of the Transporter Classification Database (TCDB) (Saier et al. 2021) revealed high similarity to proteins from the Drug:H^+^ Antiporter-2 (14 Spanner) (DHA2) family (TC# 2.A.1.3) (Table S4). The closest homologues of CpkF are found within *Streptomyces* BGCs of unsaturated polyketides such as enediynes, angucyclines, anthracyclines and polyenes (Table S5, Fig. 3).

Structurally, CpkF consists of 14 transmembrane α-helical segments, as predicted by the web server TOPCONS (Tsirigos et al. 2015), for membrane protein topology consensus prediction. The locations of the TMSs together with the hydropathy and amphipacity plots (Zhai and Saier 2001) are shown in Fig. S1. Seven conserved sequence motifs shared by MFS members and the DHA2 family can be found in the CpkF sequence (Table S6, Fig. S1). CpkF has a leucine residue (L320) in the position corresponding to D323 of QacA, an efflux pump from *Staphylococcus aureus* that confers resistance to a variety of both mono- and divalent lipophilic cationic antimicrobial compounds. Aspartic acid in TMS10 (D323) was shown by mutational studies to be necessary for QacA to accommodate divalent cations (Paulsen et al. 1996a). CpkF does not contain any acidic residues within TMS10 or TMS12, which could potentially facilitate the binding of divalent cations in the absence of the crucial Asp (Hassan et al. 2007). Accordingly, we expect CpkF to be able to transport monovalent cationic compounds. This is in line with the structure of its predicted native substrate from the coelimycin biosynthetic pathway (Fig. 1, compound 3).

**Fig. 4:**
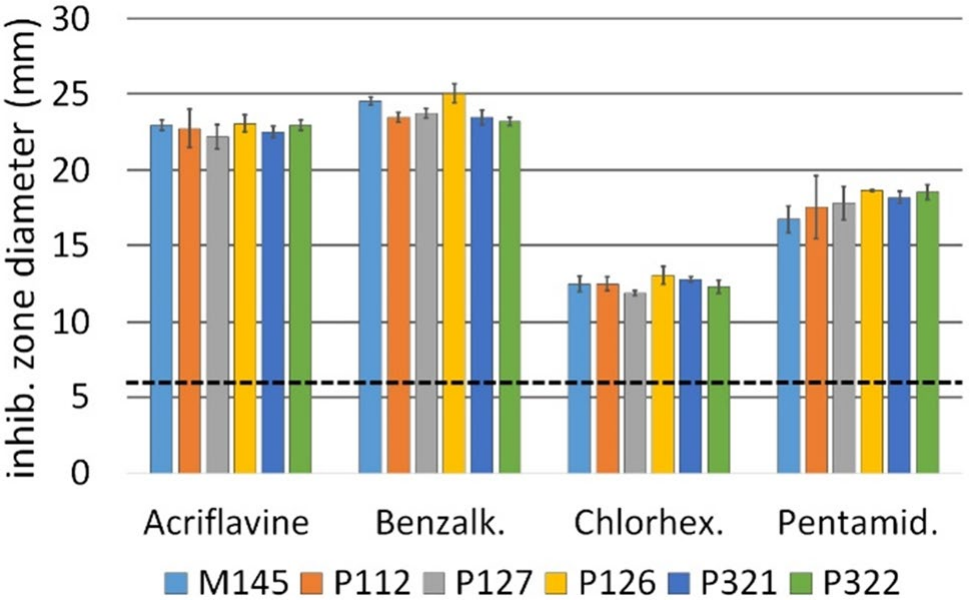
The susceptibility of *S. coelicolor* A3(2) strains to biocides (acriflavine, benzalconium, chlorhexidine and pentamidine) measured as the diameter of the growth inhibition zone. Dashed line represents the disc diameter (6 mm). The mean values and standard deviations of triplicate samples are shown. The strain names are the same as in Table 1

The susceptibilities of the wild-type *S. coelicolor* strain M145 and the *cpkF* deletion strain P112 to several compounds that are substrates of the QacA efflux pump, similar to CpkF, were compared by the disc diffusion method. Complementation strains P127 and P321, as well as their respective control strains with empty plasmids (P126 and P322), were also included in the experiments. Examples of monovalent (acriflavine and benzalconium) and divalent (chlorhexidine and pentamidine) lipophilic cationic compounds were tested. No significant differences were found between the strains (Fig. 4). This result indicated the high specificity of CpkF despite its similarity to multidrug efflux pumps.

### The intracellular coelimycin precursor(s) takes part in a negative regulatory feedback loop

**Fig. 5:**
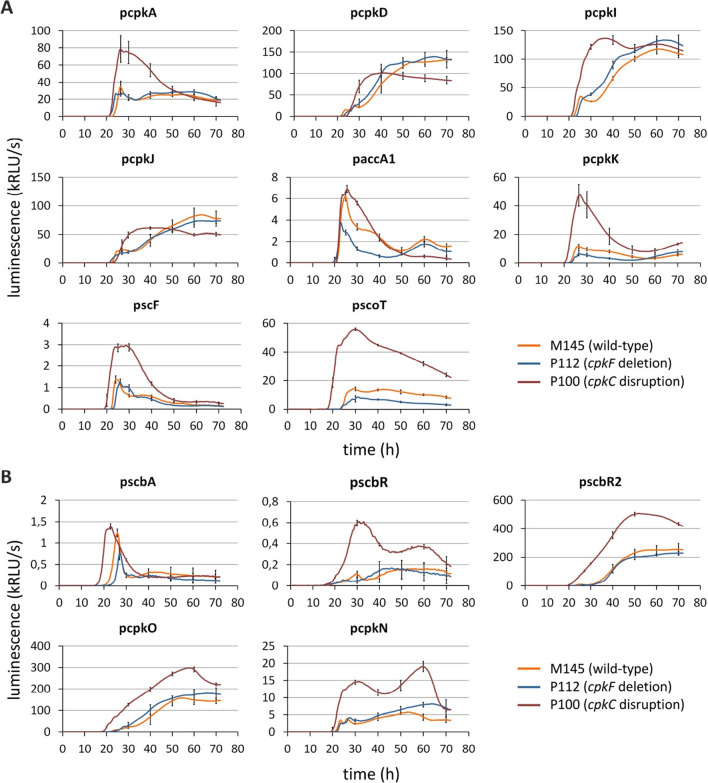
Transcription profiles of *cpk* cluster biosynthetic (**A**) and regulatory (**B**) genes. Promoter activities were measured in a luciferase-based reporter system. The wild-type (M145), *cpkF* deletion (P112) and *cpkC* disruption (P100) strains were grown on solid 79NG medium. For clarity, the standard deviation is shown for every 10 h. The legend and axes descriptions refer to all charts

To assess the effect of the lack of extracellular CPK production on the CPK BGC expression in the transporter-deficient strain, the activities of 13 promoter regions were compared between the wild-type (M145) and the *cpkF* deletion (P112) strains. A luciferase reporter system was used as described previously (Bednarz et al. 2021). Figure 5 shows that the transcription of most of the biosynthetic genes in the strain lacking CpkF remained at the same level as that in the parental strain. Similarly, the production of the activators CpkO and CpkN, the repressors ScbR and ScbR2, and the GBL synthase ScbA was not significantly affected. The only observed difference was an approximately twofold reduction in the transcription of *cpkK*, *accA1* which are involved in the substrate (malonyl-CoA) supply, and *scoT*, which encodes a type II editing thioesterase.

To address the question of whether the intracellular precursor(s) of CPK could affect the *cpk* gene cluster transcription, the activities of the same promoters were measured in the *cpkC* disruption strain (P100), which is unable to finish the synthesis of the polyketide backbone of CPK due to the lack of the functional CpkC subunit of the polyketide synthase (Fig. 1). Transcription of the whole CPK BGC was enhanced in this strain (Fig. 5).

## Discussion

The investigation of SM exporters can reveal the details of BGCs regulation by their cognate products. For example, the deletion of *drrAB* genes of the daunorubicin/doxorubicin exporter providing the antibiotic resistance to the producer, *Streptomyces peucetius*, uncovered the existence of a feedback transcriptional regulatory mechanism, where the lack of the transporter leads to the reduction of antibiotic production. Daunorubicin remaining in the cell intercalates in the DNA in a specific site and thus breaks the activatory cascade (Srinivasan et al. 2010). In *S. coelicolor* A3(2), two transporters, ActA and ActB, play a key role in a sophisticated two-step feed-forward mechanism of actinorhodin (ACT) production control. ACT biosynthetic intermediates induce *actAB* expression to make sure that the efflux pumps are in place to remove the antibiotic as soon as it is produced. The extracellular ACT is a signal by which the producing subpopulation of cells induces sustained *actAB* expression in the rest of mycelium and protects the whole culture from antimicrobial effects of the accumulating product (Xu et al. 2012).

The present study focuses on investigating the role of coelimycin polyketide in the autoregulation of its BGC. Our study model consisted of two *S. coelicolor* A3(2) mutant strains: P112 (*ΔcpkF*) deficient in extracellular CPK production due to the lack of the transporter CpkF and P100 (*ΔcpkC*) with completely abolished production of the metabolite due to the lack of the functional CpkC subunit. This allowed us to track down which CPK compounds, present in the cytoplasm — preCPK, or outside of the cell — abCPK/yCPK, could possibly have a signalling role in the cluster autoregulation.

The existence of a negative feedback loop exerted by the Cpk synthase product(s) was proposed in our previous article (Bednarz et al. 2021). It was based on the observed upregulation of CPK BGC, including the main activator CpkO, in the *cpkN* disruption strain (*ΔcpkN*). We considered it unlikely for CpkN to act as a repressor of the *cpkO* gene. Both CpkN and CpkO belong to the SARP (*Streptomyces* antibiotic regulatory proteins) family of regulators acting mostly as activators (Yan and Xia 2024) and they are both required for CPK production. CpkN is a key activator of *scoT* gene (Bednarz et al. 2021) encoding the editing thioesterase, necessary for CPK production, which works as a “cleaner” removing non-reactive acyl residues blocking the “assembly line” (Kotowska et al. 2014). Thus, *ΔcpkN* cannot produce CPK due to the lack of ScoT thioesterase. Indeed, the inability to produce CPK by this strain was reversed by providing ScoT expression from the ermEp* promoter. In the *cpkO* deletion strain (*ΔcpkO*), transcription of biosynthetic genes was obviously silenced due to the lack of the main activator — CpkO, but interestingly transcription from the native *cpkO* promoter was strongly enhanced. Therefore, we postulated that it was the lack of coelimycin synthesis that resulted in *cpkO* gene upregulation in both *ΔcpkO* and *ΔcpkN* strains, and that the activation of the remaining *cpk* genes in *ΔcpkN* was a consequence of the increased level of CpkO.

**Fig. 6:**
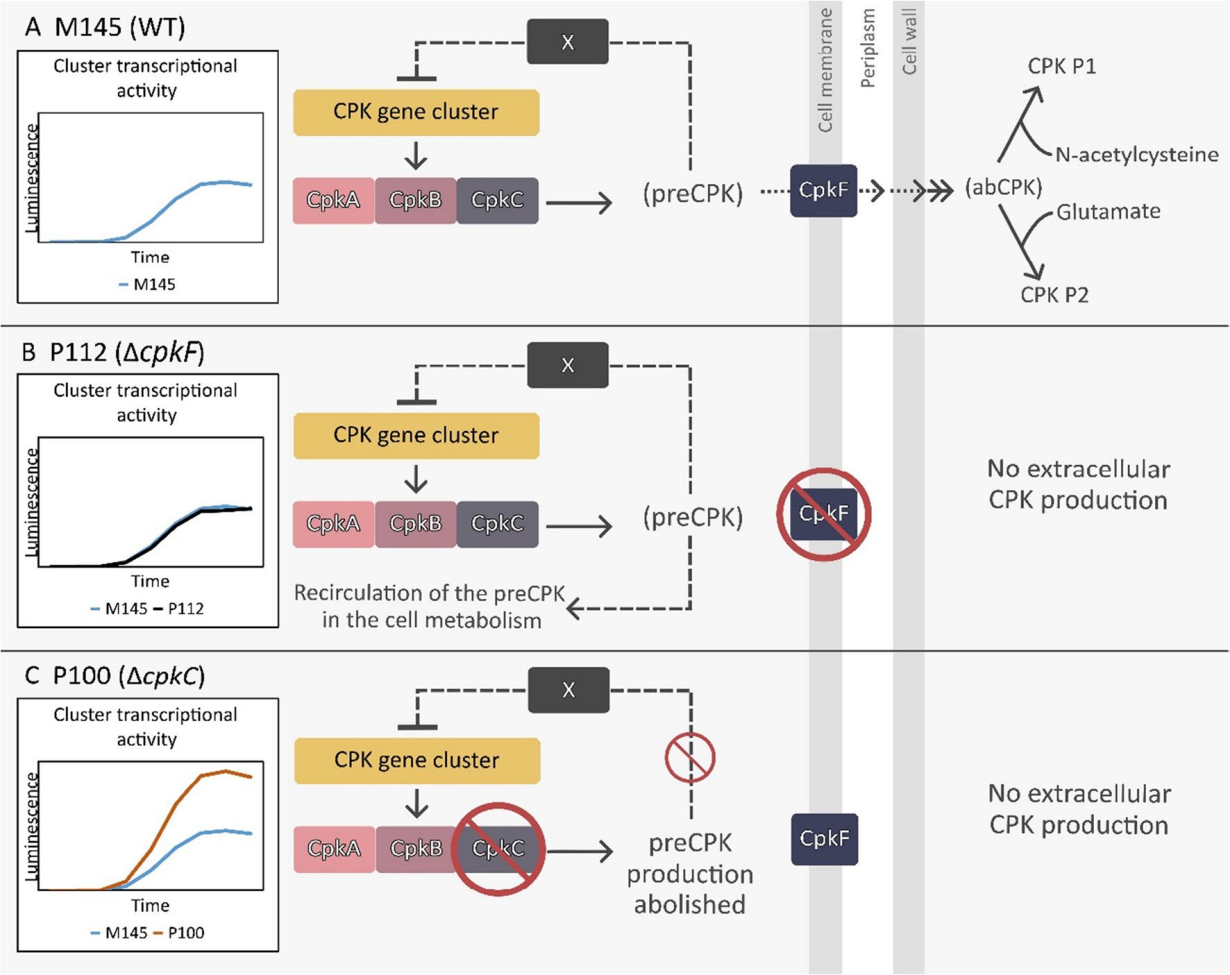
Autoregulation of the coelimycin biosynthetic gene cluster by its intracellular polyketide product. X represents an unknown receptor of preCPK ligand. (**A**) In native conditions the preCPK compound(s) acts as a negative feedback autoregulator. (**B**) Without the specific exporter CpkF intracellular precursors remain in the cytoplasm. The cluster transcription is similar to the wild-type strain with few exceptions of half-downregulated promoters indicating higher preCPK levels in the cell. (**C**) Loss of the CpkC synthase subunit leads to complete elimination of the CPK production. The cluster transcription is strongly upregulated in comparison to the WT strain

In the current study the in vivo luciferase reporter system was utilized to measure the activity of most of the CPK BGC promoters in P100 (*ΔcpkC*) and P112 (*ΔcpkF*) strains. Obtained results let us conclude that the CPK cluster is negatively regulated by the intracellular preCPK compound(s), while the extracellular CPK versions do not exert this feedback role (Fig. 6). Upregulation of all tested promoters was observed in the P100 strain unable to synthesize preCPK. The lack of the transporter CpkF did not change markedly the transcription of *cpk* genes, even though the extracellular yCPK was not produced. Synthesis of preCPK in the P112 strain seems to be undisturbed.

The observation of residual ~ 2% of CPK P2 production by the *ΔcpkF* strain indicates that a small amount of the precursor found its way outside the cells and was converted into the final product. This means that the whole machinery of initial biosynthetic steps is working properly. The preCPK compounds are present in the cell and their regulatory roles are fulfilled, which is reflected in the results of the reporter assay of the *cpk* gene promoters. We have shown that the *cpkF* gene deletion does not significantly affect the expression of biosynthetic and regulatory genes of CPK BGC (Fig. 5). An approximately twofold reduction in *cpkK*, *accA1* and *scoT* promoters activity is unlikely to drastically reduce the production of preCPK. It is not clear if the precursor accumulates in the cell or is directed to other metabolic pathways, yet the observation of lowered *cpkK*, *accA1* and *scoT* expression levels may suggest increased preCPK level in the cytoplasm and the negative regulation of the cluster. These three promoters could be the most susciptible to the metabolite-dependent regulation.

There are two possible explanations of preCPK release without CpkF. The CPK production time is correlated with extensive mycelium rebuilding (Yagüe et al. 2013). The cellular envelopes integrity could have been disturbed by naturally occurring mechanical tensions or the damage was done during the sample preparation for the HPLC measurements. Some portion of preCPK could have simply leaked out of the cell. The other possibility is that the precursor was released by another efflux pump. A BLAST search against *S. coelicolor* A3(2) proteins revealed several MFS transporters with high sequence homology to CpkF (Table S7). However, given the rather undisturbed supply of preCPK and negligible CPK P2 production in the P112 strain, these transporters cannot efficiently replace CpkF. This may be caused by their different substrate specificities and/or different time and level of expression.

The CpkF transporter seems to be dedicated to its specific substrate, despite its sequence similarity to multidrug efflux pumps such as QacA and QacB from *S. aureus*. Its deletion did not alter the susceptibility to biocides (Fig. 4), as opposed to other MFS efflux pumps, for example SCO4121 from *S. coelicolor* A3(2) (Nag and Mehra 2021).

We suspect that preCPK can influence the transcription activity of the *cpk* cluster by acting as a ligand for a yet unidentified transcription factor denoted in Fig. 6 as an “X” protein. There are two groups of cluster-encoded regulators that hypothetically could exert the role of a CPK intermediate sensor. The first ones are TetR-family repressors ScbR and ScbR2, and the second ones are SARP activators CpkO and CpkN.

It is well known that TetR-family proteins can bind low molecular ligands including products of their own BGCs (Takahashi et al. 1986). ScbR2 was shown by EMSA to dissociate from its target DNA in the presence of unecylprodigiosin and ACT (Xu et al. 2010). Considering the possibility that ScbR2 would be the preCPK-binding protein working in a high concentration of the metabolite(s), when a certain level of it is produced, it would have to increase its DNA-binding after interaction with the ligand(s), to make this regulation model work.

Recently, it has been reported for the first time that SARPs can interact with biosynthetic intermediates or final products. Nosiheptide (NOS) BGC of *Streptomyces actuosus* is activated by the only cluster-situated regulator — NosP — belonging to SARPs. It has been shown that NosP dissociates from its target promoter sequence in the presence of NOS and its intermediate (Li et al. 2018). Moreover, upon the deletion of NosP, all biosynthetic genes were silenced, while transcription from the native *nosP* promoter was enhanced. This effect resembles *cpkO* upregulation in *ΔcpkO* and *ΔcpkN* strains (Bednarz et al. 2021). In *S. actuosus* mutant strain unable to produce the raw peptide precursor of NOS, upregulation of biosynhetic genes was observed, similarly as in the *ΔcpkC* strain (P100) described here. CPK cluster-situated SARP activators, CpkO and CpkN, could be possible sensors of preCPK. PreCPK could disturb SARP binding to DNA, thus autorepressing *cpk* cluster. CpkN, just as NosP, belongs to small SARPs composed of only two domains: N-terminal DNA-binding domain and C-terminal bacterial transcriptional activation domain (BTAD). CpkO belongs to medium SARPs which contain additionally a highly conserved nucleotide-binding domain NB-ARC (Yan and Xia 2024).

It cannot be excluded that the true preCPK-binding protein is none of the so far mentioned ones. CPK BGC regulation is a highly intricate network, which was presented by our group in the past years (Bednarz et al. 2019, 2021). Regulators encoded outside of the cluster, such as response regulators from two-component systems could potentially bind preCPK as well. The *cpk* cluster encodes also an orphan histidine kinase CpkM (SCO6268), formerly OrfB, the expression pattern of which correlates with the other *cpk* genes. CpkM is a positive regulator of the cluster (Wenecki, unpublished), yet its interactome is unknown. It demonstrates that there might be plenty of so-far unidentified regulators, the presence of which is yet to be unraveled.

The transporters encoded within SM BGCs are usually assumed to export the SM out of the cell; however, their exact roles in the final steps of the natural product synthesis, as well as the effects of export on the BGC regulation and on the host self-resistance, remain underexplored (Severi and Thomas 2019). One of the strategies to avoid suicide by antibiotic-producing cells is to use a biosynthetic intermediate to induce transcription of the transporter gene so that the efflux pump is in place to remove the toxic compound as soon as it is produced (Van Keulen and Dyson 2014). Although it is generally assumed that secondary metabolite biosynthesis is completed within the cell, there is evidence for the extracellular location of some biosynthetic enzymes (Chater et al. 2010). This is the case in coelimycin biosynthesis, where the polyene intermediate must be exported to make it available to the extracellular flavin-dependent epoxidases/dehydrogenases ScF, CpkD, and CpkH, which perform post-polyketide modifications, including double epoxidation, which is responsible for the toxicity of the compound (Gomez-Escribano et al. 2012). Microarray transcriptomic analysis of *S. coelicolor* A3(2) (Nieselt et al. 2010) revealed a strong transient peak in the expression of CPK BGC at approximately 24 h. Later, the genes directly related to the synthesis of the CPK polyketide backbone (*cpkA/B/C*) are switched down to low level expression, while the transcription of genes associated with post-polyketide modifications remains constantly elevated. The common transcription profile of *cpkF* and other biosynthetic genes (particularly co-transcription with *cpkE*, *cpkG* and one of the extracellular enzymes, *cpkD*) suggests its involvement in the export of the polyketide produced by Cpk synthase and fine synchronization of the intermediate export with further enzymatic steps of biosynthesis (Fig. S2). We have shown here that the so far univestigated MFS efflux pump CpkF is indeed involved in CPK export from the cell and is necessary for the production of the final compound (Fig. 2).

To find clues about the roles of CpkF homologues in secondary metabolism, an amino acid sequence similarity search was performed against all proteins belonging to 2500 BGCs collected in the MIBiG repository (Medema et al. 2015) . The transporters most similar to CpkF were found to be involved in the biosynthesis of enediynes and other polyketides, such as angucyclines, anthracyclines and polyene macrolactams (Fig. 3). Although the final structures of the found compounds differ markedly from each other and from that of coelimycin P1, the presence of multiple conjugated double (and triple, in the case of enediynes) bonds is their common feature, which resembles the predicted polyene CPK precursor transported by CpkF. The unsaturated bonds of polyketide backbones are often involved in cyclization, oxidation and other reactions (Nett and Moore 2009; Gomez-Escribano et al. 2012; Ma et al. 2017). We consider it likely that CpkF homologues are reponsible for the export of biosynthetic intermediates, which are then modified by extracellular enzymes to yield the final product. The extracellular location of proteins can be predicted by the presence of signal peptides, short N-terminal sequences that direct newly synthesized proteins to secretion machinery and are subsequently cleaved off by specialized proteases (Almagro Armenteros et al. 2019). Indeed, analysis by SignalP software revealed the presence of proteins with signal peptides in the majority of BGCs containing CpkF homologues. A CpkD homologue was found in the enediyne sporolide biosynthetic cluster (BGC0000150, protein Strop_2710, ABP55154). ScF and CpkH homologue was found in the tetrahydroisoquinoline alkaloid naphthyridinomycin biosynthetic cluster (BGC0000394, protein AGD80628).

In addition, we recommend the use of solid Glu-MM medium and the “freeze/thaw/centrifuge” procedure as a simple and reproducible method of sample preparation for HPLC analysis of CPK P2 production. Supplementation of the minimal medium with glutamate has been shown previously to promote the conversion of CPK precursors to CPK P2 and to facilitate its direct detection in the culture medium (Kotowska et al. 2014). The use of plate cultures allowed us to overcome the notorious variability of antibiotic production rates in liquid shake cultures, which is a problem well known to investigators of *Streptomyces* physiology (Siebenberg et al. 2010).

## Supplementary Information

Below is the link to the electronic supplementary material.

Supplementary file1 (PDF 592 KB)

## Data Availability

All data supporting the findings of this study are available within the paper and its Supplementary Information. Original files can be made available upon reasonable request to the authors.

## References

[CR1] Almagro Armenteros JJ, Tsirigos KD, Sønderby CK, Petersen TN, Winther O, Brunak S, von Heijne G, Nielsen H (2019) SignalP 5.0 improves signal peptide predictions using deep neural networks. Nat Biotechnol 37:420–423. 10.1038/S41587-019-0036-Z 30778233 10.1038/s41587-019-0036-z

[CR2] Altschul SF, Gish W, Miller W, Myers EW, Lipman DJ (1990) Basic local alignment search tool. J Mol Biol 215:403–410. 10.1016/S0022-2836(05)80360-2 2231712 10.1016/S0022-2836(05)80360-2

[CR3] Awodi UR, Ronan JL, Masschelein J, De Los Santos ELC, Challis GL (2016) Thioester reduction and aldehyde transamination are universal steps in actinobacterial polyketide alkaloid biosynthesis. Chem Sci 8. 10.1039/c6sc02803a 10.1039/c6sc02803a PMC5365063 28451186

[CR4] Bednarz B, Kotowska M, Pawlik KJ (2019) Multi-level regulation of coelimycin synthesis in *Streptomyces coelicolor* A3(2). Appl Microbiol Biotechnol 103:6423–6434. 10.1007/s00253-019-09975-w 31250060 10.1007/s00253-019-09975-w PMC6667686

[CR5] Bednarz B, Millan-Oropeza A, Kotowska M, Świat M, Quispe Haro JJ, Henry C, Pawlik K (2021) Coelimycin Synthesis Activatory Proteins Are Key Regulators of Specialized Metabolism and Precursor Flux in *Streptomyces coelicolor* A3(2). Front Microbiol 12. 10.3389/FMICB.2021.616050 10.3389/fmicb.2021.616050 PMC8062868 33897632

[CR7] Chater KF, Biró S, Lee KJ, Palmer T, Schrempf H (2010) The complex extracellular biology of *Streptomyces*. FEMS Microbiol Rev 34:171–198. 10.1111/J.1574-6976.2009.00206.X 20088961 10.1111/j.1574-6976.2009.00206.x

[CR9] Drew D, North RA, Nagarathinam K, Tanabe M (2021) Structures and General Transport Mechanisms by the Major Facilitator Superfamily (MFS). Chem Rev 121:5289–5335. 10.1021/ACS.CHEMREV.0C00983 33886296 10.1021/acs.chemrev.0c00983 PMC8154325

[CR10] Getsin I, Nalbandian GH, Yee DC, Vastermark A, Paparoditis PCG, Reddy VS, Saier MH (2013) Comparative genomics of transport proteins in developmental bacteria: *Myxococcus xanthus* and *Streptomyces coelicolor*. BMC Microbiol 13. 10.1186/1471-2180-13-279 10.1186/1471-2180-13-279 PMC3924187 24304716

[CR11] Gomez-Escribano JP, Song L, Fox DJ, Yeo V, Bibb MJ, Challis GL (2012) Structure and biosynthesis of the unusual polyketide alkaloid coelimycin P1, a metabolic product of the cpk gene cluster of *Streptomyces coelicolor* M145. Chem Sci 3:2716–2720. 10.1039/C2SC20410J

[CR12] Gottelt M, Kol S, Gomez-Escribano JP, Bibb M, Takano E (2010) Deletion of a regulatory gene within the cpk gene cluster reveals novel antibacterial activity in *Streptomyces coelicolor* A3(2). Microbiology (n y) 156:2343–2353. 10.1099/mic.0.038281-0 10.1099/mic.0.038281-0 20447997

[CR13] Gust B, Chandra G, Jakimowicz D, Yuqing T, Bruton CJ, Chater KF (2004) λ Red-Mediated Genetic Manipulation of Antibiotic-Producing *Streptomyces*. Adv Appl Microbiol 54:107–128. 10.1016/S0065-2164(04)54004-2 15251278 10.1016/S0065-2164(04)54004-2

[CR14] Hassan KA, Skurray RA, Brown MH (2007) Transmembrane helix 12 of the *Staphylococcus aureus* multidrug transporter QacA lines the bivalent cationic drug binding pocket. J Bacteriol 189:9131–9135. 10.1128/JB.01492-07 17951386 10.1128/JB.01492-07 PMC2168635

[CR15] Kieser T, Bibb MJ, Buttner MJ, Chater KF, Hopwood DA (2000) Practical *Streptomyces* Genetics. John Innes Centre Ltd. 529

[CR16] Kotowska M, Ciekot J, Pawlik K (2014) Type II thioesterase ScoT is required for coelimycin production by the modular polyketide synthase Cpk of *Streptomyces coelicolor* A3(2). Acta Biochim Pol 61:141–147. 10.18388/ABP.2014_1936 24660171

[CR17] Kumar S, Lekshmi M, Parvathi A, Ojha M, Wenzel N, Varela MF (2020) Functional and Structural Roles of the Major Facilitator Superfamily Bacterial Multidrug Efflux Pumps. Microorganisms 8. 10.3390/MICROORGANISMS8020266 10.3390/microorganisms8020266 PMC7074785 32079127

[CR18] Kutas P, Feckova L, Rehakova A, Novakova R, Homerova D, Mingyar E, Rezuchova B, Sevcikova B, Kormanec J (2013) Strict control of auricin production in *Streptomyces aureofaciens* CCM 3239 involves a feedback mechanism. Appl Microbiol Biotechnol 97:2413–2421. 10.1007/s00253-012-4505-2 23081778 10.1007/s00253-012-4505-2

[CR21] Letunic I, Bork P (2021) Interactive Tree Of Life (iTOL) v5: an online tool for phylogenetic tree display and annotation. Nucleic Acids Res 49:W293–W296. 10.1093/NAR/GKAB301 33885785 10.1093/nar/gkab301 PMC8265157

[CR22] Li J, Li Y, Niu G, Guo H, Qiu Y, Lin Z, Liu W, Tan H (2018) NosP-Regulated Nosiheptide Production Responds to Both Peptidyl and Small-Molecule Ligands Derived from the Precursor Peptide. Cell Chem Biol 25:143-153.e4. 10.1016/j.chembiol.2017.10.012 29198568 10.1016/j.chembiol.2017.10.012

[CR23] Li Q, Wang L, Xie Y, Wang S, Chen R, Hong B (2013) SsaA, a member of a novel class of transcriptional regulators, controls sansanmycin production in *Streptomyces sp*. strain SS through a feedback mechanism. J Bacteriol 195:2232–2243. 10.1128/JB.00054-13 23475969 10.1128/JB.00054-13 PMC3650532

[CR24] Li X, Yu T, He Q, McDowall KJ, Jiang B, Jiang Z, Wu L, Li G, Li Q, Wang S, Shi Y, Wang L, Hong B (2015) Binding of a biosynthetic intermediate to AtrA modulates the production of lidamycin by *Streptomyces globisporus*. Mol Microbiol 96:1257–1271. 10.1111/mmi.13004 25786547 10.1111/mmi.13004

[CR25] Lorca GL, Barabote RD, Zlotopolski V, Tran C, Winnen B, Hvorup RN, Stonestrom AJ, Nguyen E, Huang LW, Kim DS, Saier MH (2007) Transport capabilities of eleven gram-positive bacteria: comparative genomic analyses. Biochim Biophys Acta 1768:1342–1366. 10.1016/J.BBAMEM.2007.02.007 17490609 10.1016/j.bbamem.2007.02.007 PMC2592090

[CR26] Ma SY, Xiao YS, Zhang B, Shao FL, Guo ZK, Zhang JJ, Jiao RH, Sun Y, Xu Q, Tan RX, Ge HM (2017) Amycolamycins A and B, Two Enediyne-Derived Compounds from a Locust-Associated Actinomycete. Org Lett 19:6208–6211. 10.1021/ACS.ORGLETT.7B03113/SUPPL_FILE/OL7B03113_SI_001.PDF 29090939 10.1021/acs.orglett.7b03113

[CR27] Medema MH, Kottmann R, Yilmaz P, Cummings M, Biggins JB, Blin K, De Bruijn I, Chooi YH, Claesen J, Coates RC, Cruz-Morales P, Duddela S, Düsterhus S, Edwards DJ, Fewer DP, Garg N, Geiger C, Gomez-Escribano JP, Greule A, Hadjithomas M, Haines AS, Helfrich EJN, Hillwig ML, Ishida K, Jones AC, Jones CS, Jungmann K, Kegler C, Kim HU, Kötter P, Krug D, Masschelein J, Melnik AV, Mantovani SM, Monroe EA, Moore M, Moss N, Nützmann HW, Pan G, Pati A, Petras D, Reen FJ, Rosconi F, Rui Z, Tian Z, Tobias NJ, Tsunematsu Y, Wiemann P, Wyckoff E, Yan X, Yim G, Yu F, Xie Y, Aigle B, Apel AK, Balibar CJ, Balskus EP, Barona-Gómez F, Bechthold A, Bode HB, Borriss R, Brady SF, Brakhage AA, Caffrey P, Cheng YQ, Clardy J, Cox RJ, De Mot R, Donadio S, Donia MS, Van Der Donk WA, Dorrestein PC, Doyle S, Driessen AJM, Ehling-Schulz M, Entian KD, Fischbach MA, Gerwick L, Gerwick WH, Gross H, Gust B, Hertweck C, Höfte M, Jensen SE, Ju J, Katz L, Kaysser L, Klassen JL, Keller NP, Kormanec J, Kuipers OP, Kuzuyama T, Kyrpides NC, Kwon HJ, Lautru S, Lavigne R, Lee CY, Linquan B, Liu X, Liu W, Luzhetskyy A, Mahmud T, Mast Y, Méndez C, Metsä-Ketelä M, Micklefield J, Mitchell DA, Moore BS, Moreira LM, Müller R, Neilan BA, Nett M, Nielsen J, O’Gara F, Oikawa H, Osbourn A, Osburne MS, Ostash B, Payne SM, Pernodet JL, Petricek M, Piel J, Ploux O, Raaijmakers JM, Salas JA, Schmitt EK, Scott B, Seipke RF, Shen B, Sherman DH, Sivonen K, Smanski MJ, Sosio M, Stegmann E, Süssmuth RD, Tahlan K, Thomas CM, Tang Y, Truman AW, Viaud M, Walton JD, Walsh CT, Weber T, Van Wezel GP, Wilkinson B, Willey JM, Wohlleben W, Wright GD, Ziemert N, Zhang C, Zotchev SB, Breitling R, Takano E, Glöckner FO (2015) Minimum Information about a Biosynthetic Gene cluster. Nat Chem Biol 11:625–631. 10.1038/NCHEMBIO.1890 26284661 10.1038/nchembio.1890 PMC5714517

[CR28] Nag A, Mehra S (2021) A Major Facilitator Superfamily (MFS) Efflux Pump, SCO4121, from *Streptomyces coelicolor* with Roles in Multidrug Resistance and Oxidative Stress Tolerance and Its Regulation by a MarR Regulator. Appl Environ Microbiol 87:1–23. 10.1128/AEM.02238-20 10.1128/AEM.02238-20 PMC8091613 33483304

[CR29] Nett M, Moore BS (2009) Exploration and engineering of biosynthetic pathways in the marine actinomycete *Salinispora tropica*. In: Pure and Applied Chemistry. pp 1075–1084

[CR30] Nieselt K, Battke F, Herbig A, Bruheim P, Wentzel A, Jakobsen ØM, Sletta H, Alam MT, Merlo ME, Moore J, Omara WAM, Morrissey ER, Juarez-Hermosillo MA, Rodríguez-García A, Nentwich M, Thomas L, Iqbal M, Legaie R, Gaze WH, Challis GL, Jansen RC, Dijkhuizen L, Rand DA, Wild DL, Bonin M, Reuther J, Wohlleben W, Smith MCM, Burroughs NJ, Martín JF, Hodgson DA, Takano E, Breitling R, Ellingsen TE, Wellington EMH (2010) The dynamic architecture of the metabolic switch in *Streptomyces coelicolor*. BMC Genomics 11. 10.1186/1471-2164-11-10

[CR31] Paulsen IT, Brown MH, Littlejohn TG, Mitchell BA, Skurray RA (1996a) Multidrug resistance proteins QacA and QacB from *Staphylococcus aureus*: membrane topology and identification of residues involved in substrate specificity. Proc Natl Acad Sci U S A 93:3630. 10.1073/PNAS.93.8.3630 8622987 10.1073/pnas.93.8.3630 PMC39662

[CR32] Paulsen IT, Brown MH, Skurray RA (1996b) Proton-dependent multidrug efflux systems. Microbiol Rev 60:575–608. 10.1128/MR.60.4.575-608.1996 8987357 10.1128/mr.60.4.575-608.1996 PMC239457

[CR33] Pawlik K, Kotowska M, Chater KF, Kuczek K, Takano E (2007) A cryptic type I polyketide synthase (cpk) gene cluster in *Streptomyces coelicolor* A3(2). Arch Microbiol 187:87–99. 10.1007/S00203-006-0176-7/FIGURES/3 17009021 10.1007/s00203-006-0176-7

[CR34] Pawlik K, Kotowska M, Kolesiński P (2010) *Streptomyces coelicolor* A3(2) Produces a New Yellow Pigment Associated with the Polyketide Synthase Cpk. Microb Physiol 19:147–151. 10.1159/000321501 10.1159/000321501 20924201

[CR37] Saier MH, Reddy VS, Moreno-Hagelsieb G, Hendargo KJ, Zhang Y, Iddamsetty V, Lam KJK, Tian N, Russum S, Wang J, Medrano-Soto A (2021) The Transporter Classification Database (TCDB): 2021 update. Nucleic Acids Res 49:D461–D467. 10.1093/NAR/GKAA1004 33170213 10.1093/nar/gkaa1004 PMC7778945

[CR38] Sambrook J, W Russell D (2001) Molecular Cloning: A Laboratory Manual. Cold Spring Harbor Laboratory Press, Cold Spring Harbor, NY 999. 10.1016/0092-8674(90)90210-6

[CR39] Severi E, Thomas GH (2019) Antibiotic export: transporters involved in the final step of natural product production. Microbiology (Reading) 165:805–818. 10.1099/MIC.0.000794 30964430 10.1099/mic.0.000794

[CR40] Siebenberg S, Bapat PM, Lantz AE, Gust B, Heide L (2010) Reducing the variability of antibiotic production in *Streptomyces* by cultivation in 24-square deepwell plates. J Biosci Bioeng 109:230–234. 10.1016/J.JBIOSC.2009.08.479 20159569 10.1016/j.jbiosc.2009.08.479

[CR41] Sievers F, Wilm A, Dineen D, Gibson TJ, Karplus K, Li W, Lopez R, McWilliam H, Remmert M, Söding J, Thompson JD, Higgins DG (2011) Fast, scalable generation of high-quality protein multiple sequence alignments using Clustal Omega. Mol Syst Biol 7. 10.1038/MSB.2011.75 10.1038/msb.2011.75 PMC3261699 21988835

[CR42] Srinivasan P, Palani SN, Prasad R (2010) Daunorubicin efflux in *Streptomyces peucetius* modulates biosynthesis by feedback regulation. FEMS Microbiol Lett 305:18–27. 10.1111/j.1574-6968.2010.01905.x 20158521 10.1111/j.1574-6968.2010.01905.x

[CR43] Takahashi M, Altschmied L, Hillen W (1986) Kinetic and equilibrium characterization of the Tet repressor-tetracycline complex by fluorescence measurements. Evidence for divalent metal ion requirement and energy transfer. J Mol Biol 187:341–348. 10.1016/0022-2836(86)90437-7 3517354 10.1016/0022-2836(86)90437-7

[CR44] Tsirigos KD, Peters C, Shu N, Käll L, Elofsson A (2015) The TOPCONS web server for consensus prediction of membrane protein topology and signal peptides. Nucleic Acids Res 43:W401–W407. 10.1093/NAR/GKV485 25969446 10.1093/nar/gkv485 PMC4489233

[CR45] Van Keulen G, Dyson PJ (2014) Production of specialized metabolites by *Streptomyces coelicolor* A3(2). Adv Appl Microbiol 89:217–266. 10.1016/B978-0-12-800259-9.00006-8 25131404 10.1016/B978-0-12-800259-9.00006-8

[CR46] Wang SC, Davejan P, Hendargo KJ, Javadi-Razaz I, Chou A, Yee DC, Ghazi F, Lam KJK, Conn AM, Madrigal A, Medrano-Soto A, Saier MH (2020) Expansion of the Major Facilitator Superfamily (MFS) to include novel transporters as well as transmembrane-acting enzymes. Biochimica et Biophysica Acta (BBA) - Biomembranes 1862:183277. 10.1016/J.BBAMEM.2020.183277 10.1016/j.bbamem.2020.183277 PMC7939043 32205149

[CR47] Xu G, Wang J, Wang L, Tian X, Yang H, Fan K, Yang K, Tan H (2010) “Pseudo” γ-butyrolactone receptors respond to antibiotic signals to coordinate antibiotic biosynthesis. J Biol Chem 285:27440–27448. 10.1074/jbc.M110.143081 20562102 10.1074/jbc.M110.143081 PMC2930742

[CR48] Xu Y, Willems A, Au-Yeung C, Tahlan K, Nodwell JR (2012) A two-step mechanism for the activation of actinorhodin export and resistance in *Streptomyces coelicolor*. mBio 3. 10.1128/mBio.00191-12 10.1128/mBio.00191-12 PMC3482498 23073761

[CR49] Yagüe P, Rodríguez-García A, López-García MT, Martín JF, Rioseras B, Sánchez J, Manteca A (2013) Transcriptomic Analysis of Streptomyces coelicolor Differentiation in Solid Sporulating Cultures: First Compartmentalized and Second Multinucleated Mycelia Have Different and Distinctive Transcriptomes. PLoS One 8. 10.1371/journal.pone.0060665 10.1371/journal.pone.0060665 PMC3610822 23555999

[CR50] Yan Y, Xia H (2024) The roles of SARP family regulators involved in secondary metabolism in *Streptomyces*. Front Microbiol 15:1368809. 10.3389/fmicb.2024.1368809 38550856 10.3389/fmicb.2024.1368809 PMC10972967

[CR51] Zhai Y, Saier J (2001) A web-based program (WHAT) for the simultaneous prediction of hydropathy, amphipathicity, secondary structure and transmembrane topology for a single protein sequence. J Mol Microbiol Biotechnol 3:501–502 11545267

[CR52] Zhou Z, Sun N, Wu S, Li YQ, Wang Y (2016) Genomic data mining reveals a rich repertoire of transport proteins in *Streptomyces*. BMC Genomics 17(Suppl):7. 10.1186/S12864-016-2899-4 27557108 10.1186/s12864-016-2899-4 PMC5001237

