# Supplementary material for: Coelimycin inside out — negative feedback regulation by its intracellular precursors
Source: Appl Microbiol Biotechnol. 2024 Dec 10;108(1):531. doi: 10.1007/s00253-024-13366-1 (PMC11632069; doi:10.1007/s00253-024-13366-1)
Supplement: Supplementary file 1 — Supplementary file1 (PDF 592 KB) [file 253_2024_13366_MOESM1_ESM.pdf]

Supplementary Material

Coelimycin inside out - negative feedback regulation by its intracellular precursors

Magdalena Kotowska, Mateusz Wenecki, Bartosz Bednarz, Jarosław Ciekot, Wojciech Pasławski, Tomasz Buhl, Krzysztof Pawlik

TABLE S1 Oligonucleotides used in this work. Restriction sites are in bold. Lower case letters indicate sequences compatible with pFLUX and pFLUXH plasmids.

Name	Sequence 5'-3'	Restriction sites	Application
CpkF_D_Fw	CACAGGCACAGAGAACGAAAGAGGGAACCCATGTCTTCGATTCCGGGGATCCGTCGACC	-	Amplification of *hyg* cassette for *cpkF* gene deletion
CpkF_D_Rv	CCTTCAGGCGCTGGCGGCTGTAGGCTGGAGCTGCTTCGTGACGGGCTCTTCGCGGGCC	-	
CpkF_Ex_F	**GGATCCCATATG**TCTTCGAACACCGCTCCC	*Bam*HI, *Nde*I	Amplification of *cpkF* gene
CpkF_Ex_W	**GAATTC**A**AAGCTT**GGCGCTGGTGACGGGCTCTTC	*Eco*RI, *Hind*III	
CpkF_Ex_R	**GAATTC**GGCGCTGGTGACGGGCTCTTC	*Eco*RI	
GFLUX-F	gtacttcgcgaaagcttgat	-	Amplification of fragments inserted between *Hind*III and *Nde*I sites of pFLUX and pFLUXH plasmids for cloning by Gibson assembly into pFLUX and pFLUXH linearized by *Hind*III and *Nde*I
GFLUX-R	gaacgagatcttcttcgtca	-	
p6282-G1	gaccaaaggaggcggacaACGTCGATCCAGCTCGC		Amplification of *cpkI* promoter (pcpkI) for cloning by Gibson assembly into pFLUX or pFLUXH linearized with *Nde*I
p6282-G2	aacgagatcttcttcgtcatCGCGACCGCAGAAGC		
p6283-G1	gaccaaaggaggcggacaACCTTGGTGACACCGGAG		Amplification of *cpkJ* promoter (pcpkJ) for cloning by Gibson assembly into pFLUX or pFLUXH linearized with *Nde*I
p6283-G2	aacgagatcttcttcgtcatACCGCCGAGGGTAATTG		
ksTF	CGCTCACCACCGAGGAGTTCA	-	Amplification of *cpkK* promoter (pcpkK)
p6284_Nde	**CATATG**AGTCCTTTCCAGGCGAGTCCA	NdeI	
pA1-582-F	aacgagatcttcttcgtcatGCCACGATTGGCGATGAG		Amplification of *accA1* promoter (paccA1) for cloning by Gibson assembly into pFLUX or pFLUXH linearized with *Nde*I and *Bam*HI
pA1-582-R	atatcggcatcgatgcatggTTGCCGACCCATCGCTG		

TABLE S2 Plasmids and cosmids used in this study.

Plasmid/cosmid	Description	Source or reference
St1G7	SuperCos1 cosmid carrying fragment of *S. coelicolor* A3(2) chromosome encompassing part of *cpk* gene cluster including *cpkF* gene (bp 6905834 to 6947687)	http://www.strepdb.streptomyces.org.uk
St1G7-cpkF_{DM}	St1G7 cosmid derivative, in which *cpkF* gene was replaced by means of PCR-targeting with a hygromycin resistance cassette *hyg* amplified with primers CpkF_D_Fw and CpkF_D_Rv on the template of pIJ10700 plasmid linearized by digestion with *Xho*I; recombineering was performed in *E.coli* BW25113/pIJ790	this work
pIJ10700	pBluescript II KS(+) containing *hyg-oriT* cassette	(Gust et al. 2004)
pGEM-T Easy	T-vector for direct cloning of PCR products (Amp^R)	Promega
pGEM-cpkF	pGEM-T Easy with *cpkF* gene amplified with primers CpkF_Ex_Fw and CpkF_Ex_W	this work
pET-21b(+)	Expression vector for His-tagged recombinant proteins	Novagen
pWP2	pET-21b(+) plasmid with *cpkF* gene (*Nde*I-*Hind*III fragment from pGEM-cpkF)	this work
pCJW93	High copy number plasmid, $Apra^R$ (*aac3(IV)*), *oriT* (RK2), $Thio^R$ (*tsr*), promoter *tipA*	(Wilkinson et al. 2002)
pWP3	Plasmid pCJW93 in which P*tipA* promoter replaced with *ermEp**	(Pawlik et al. 2021)
pWP4	pWP3 plasmid with *cpkF* gene; Bpu1102I (blunted with S1 nuclease)-*Nde*I fragment from pWP2 plasmid was inserted into *Eco*RI (blunted with S1 nuclease) and NdeI sites of pWP3	this work
pGEX-6P-2	Expression vector for GST fusion proteins	GE Healthcare
pSS2	pGEX-6P-2 plasmid with *cpkF* gene amplified with primers CpkF_Ex_Fw and CpkF_Ex_R was inserted into *Bam*HI and *Eco*RI sites	
pIJ10257	ΦBT1 integrating overexpression plasmid containing strong constitutive promoter *ermEp**, Hyg^R	(Hong et al. 2005)
pIJ10257-cpkF	pIJ10257 plasmid with *cpkF* gene (*Nde*I-*Xho*I fragment from pSS2)	this work
pSET152	ΦC31 integrating plasmid, $Apra^R$ (*aac3(IV)*)	(Kieser et al. 2000)
pIJ10257apra	pIJ10257 plasmid in which hygromycin resistance was replaced with apramycin resistance gene. EcoRV-KpnI fragment (3505 pz) from pIJ10257 and EcoRV-BlpI fragment (2012 pz) from pSET152 were blunted with Klenow enzyme and ligated. Correct orientation of ligated fragments restored *Eco*RV restriction site.	this work
pIJ10257apra-cpkF	pIJ10257apra plasmid with *cpkF* gene (*Nde*I-*Hind*III fragment from pGEM-cpkF)	this work
pFLUX	ΦBT1 integrating reporter plasmid with a promoterless luciferase operon *luxCDAEB* and apramycin resistance cassette	(Craney et al. 2007)
pFLUX-pcpkA	Reporter plasmid pFLUX with *cpkA* promoter	this work
pFLUX-pcpkD	Reporter plasmid pFLUX with *cpkD* promoter	this work
pFLUX-pscF	Reporter plasmid pFLUX with *scF* promoter	this work
pFLUX-pscoT	Reporter plasmid pFLUX with *scoT* promoter	this work
pFLUX-pcpkN	Reporter plasmid pFLUX with *cpkN* promoter	this work
pFLUX-pcpkO	Reporter plasmid pFLUX with *cpkO* promoter	this work

pFLUX-pscbA	Reporter plasmid pFLUX with *scbA* promoter	this work
pFLUX-pscbR	Reporter plasmid pFLUX with *scbR* promoter	this work
pFLUX-pscbR2	Reporter plasmid pFLUX with *cpkR2* promoter	this work
pFLUX-pI-882	Reporter plasmid pFLUX with *cpkI* promoter	this work
pFLUX-pcpkJ	Reporter plasmid pFLUX with *cpkJ* promoter	this work
pFLUX-pcpkK	Reporter plasmid pFLUX with *cpkK* promoter	this work
pFLUX-paccA1-582	Reporter plasmid pFLUX with *accA1* promoter	this work
pFLUXH	ΦBT1 integrating reporter plasmid with a promoterless luciferase operon *luxCDAEB* and hygromycin resistance cassette	(Szafran et al. 2016)
pFLUXH-pcpkA	Reporter plasmid pFLUXH with *cpkA* promoter	(Bednarz et al. 2021)
pFLUXH-pcpkD	Reporter plasmid pFLUXH with *cpkD* promoter	(Bednarz et al. 2021)
pFLUXH-pscF	Reporter plasmid pFLUXH with *scF* promoter	(Bednarz et al. 2021)
pFLUXH-pscoT	Reporter plasmid pFLUXH with *scoT* promoter	(Bednarz et al. 2021)
pFLUXH-pcpkN	Reporter plasmid pFLUXH with *cpkN* promoter	(Bednarz et al. 2021)
pFLUXH-pcpkO	Reporter plasmid pFLUXH with *cpkO* promoter	(Bednarz et al. 2021)
pFLUXH-pscbA	Reporter plasmid pFLUXH with *scbA* promoter	(Bednarz *et al.*, 2021)
pFLUXH-pscbR	Reporter plasmid pFLUXH with *scbR* promoter	(Bednarz et al. 2021)
pFLUXH-pscbR2	Reporter plasmid pFLUXH with *scbR2* promoter	(Bednarz et al. 2021)
pFLUXH-pI-882	Reporter plasmid pFLUXH with *cpkI* promoter	this work
pFLUXH-pcpkJ	Reporter plasmid pFLUXH with *cpkJ* promoter	this work
pFLUXH-pcpkK	Reporter plasmid pFLUXH with *cpkK* promoter	this work
pFLUXH-paccA1-582	Reporter plasmid pFLUXH with *accA1* promoter	this work

TABLE S3 Bacterial strains used in this study.

**Strain**	**Relevant genotype**	**Source or reference**
Escherichia coli		
DH5α	$F^{–}$ φ80*lac*ZΔM15 Δ(*lac*ZYA-*arg*F)U169 *rec*A1 *end*A1 *hsd*R17($r_K^{–}$, m_K^{+}) *pho*A *sup*E44 $λ^{–}$*thi*-1 *gyr*A96 *rel*A1	Promega
BW25113/pIJ10790	Recombineering strain harbouring arabinose-inducible RED genes on the plasmid pIJ790 *(lacI*$^{+}$*rrnB*$_{T14}$ Δ*lacZ*$_{WJ16}$ *hsdR*514 Δ*araBAD*$_{AH33}$ Δ*rhaBAD*$_{LD78}$ *rph-1* Δ*(araB–D)567* Δ*(rhaD–B)568* Δ*lacZ4787*(::*rrnB*-3) *hsdR514 rph-1* pIJ790)	(Gust et al. 2004)
ET12567/pUZ8002	Strain for conjugal transfer of DNA from *E. coli* to *Streptomyces* (*dam dcm hsdS* Cam^R Tet^R on the bacterial chromosome; *tra* Kan^R RP4 23 on pUZ8002)	(Kieser et al. 2000)
Streptomyces coelicolor **A3(2)**		

M145	Wild type strain,<br>*S. coelicolor* A3(2) (SCP1$^-$ SCP2$^-$)	(Kieser et al. 2000)
P100	Strain unable to produce coelimycin due to *cpkC* gene disruption	(Pawlik et al. 2007)
P112 (Δ*cpkF*)	Deletion of *cpkF (SCO6278)* gene (replacement with hygromycin resistance cassette)	this work
P126	P112 strain with pWP3 plasmid (control strain)	this work
P127	P112 strain with pWP4 plasmid	this work
P321	P112 strain with pIJ10257apra-cpkF plasmid	this work
P322	P112 strain with pIJ10257apra plasmid (control strain)	this work
M145, P112 and P100 derivatives for luciferase reporter assay	The strains M145 and P112 harbouring pFLUX derivatives and P100 strain harbouring pFLUXH derivatives containing promoter sequences listed in table S2	this work

Table S4 The top-scoring homologues of the CpkF protein found by a BLAST search of the Transporter Classification Database (TCDB) (Saier et al. 2021)

Protein (TCDB acc.)	Organism	Transported compound	Length (aa)	% of ident. aa	% of similar aa	E-value	Reference
QepA (A5H8A5)	*Escherichia coli*	hydrophilic fluoroquinolones, norfloxacin, ciprofloxacin	511	47	63	2e-111	(Périchon et al. 2008)
EbrC (Q939A4)	*Streptomyces lividans*	ethidium bromide, acriflavine, tetracycline, nalidixic acid, methyltriphenyl-phosphonium, proflavin, norfloxacin	521	44	60	6e-110	(Lee et al. 2003; Lee et al. 2007)
PqrB/ SCO1567 (Q9L1B4)	*Streptomyces coelicolor*	paraquat	510	45	61	3e-105	(Cho et al. 2003)
LfrA (Q50392)	*Mycobacterium smegmatis*	ethidium bromide, acriflavine, fluoroquinolones	504	41	57	7e-92	(Rodrigues et al. 2011)
QacA (P0A0J9)	*Staphylococcus aureus*	monovalent and divalent cationic lipophilic compounds	514	34	56	2e-83	(Brown and Skurray 2001)

Table S5 The top-scoring homologues of the CpkF protein found by a BLAST search of the Minimum Information about a Biosynthetic Gene cluster (MIBiG) repository (version 3.1) (Medema et al. 2015)

Protein (GenBank acc.)	Organism	BGC (MIBiG acc.)	BGC main product	Length (aa)	% of ident. aa	% of similar aa	E-value

Strop_2718 (ABP55162)	*Salinispora tropica CNB-440*	BGC0000150	sporolide A, B	528	48	63	5e-151
(WP_018960037)	*Streptomyces* sp. *CNB091*	BGC0002010	streptophenazine B, C, F, G, H	516	48	65	7e-145
PgaJ2 (AHW57775)	*Streptomyces* sp. *PGA64*	BGC0000262	gaudimycin A, C, D, prejadomycin, rabelomycin, UWM6	507	48	64	3e-138
May1 (AVO00800)	*Streptomyces* sp.	BGC0001661	mayamycin	550	48	65	3e-137
NapR4 (AGD80627)	*Streptomyces lusitanus*	BGC0000394	naphthyridinomycin	529	48	65	3e-135
Strop_2735 (ABP55179)	*Salinispora tropica CNB-440*	BGC0000150	sporolide A, B	481	51	67	4e-133
BecN (ACO94466)	*Streptomyces* sp. *DSM 21069*	BGC0000029	BE-14106	524	44	63	1e-132
(ANY94442)	*Streptomyces* sp. *CB02366*	BGC0001584	C-1027	521	46	62	6e-131
KedX (AFV52189)	*Streptoalloteichus* sp. *ATCC 53650*	BGC0000081	kedarcidin	560	49	65	2e-129
HrnN (SAI82906)	*Streptomyces* sp. *CMB-0406 CBM-0406*	BGC0002101	heronamide A, B, C	523	44	62	5e-129
mlaN (ACO94494)	*Streptomyces* sp. *MP39-85*	BGC0000097	ML-449	523	43	62	6e-128
SgcB (ALU98434)	*Streptomyces globisporus C-1027*	BGC0001397	lidamycin	521	45	62	2e-126
SgcB (AAL06672)	*Streptomyces globisporus*	BGC0000965	C-1027	521	45	62	2e-126
AcmR1 (ATV95594)	*Amycolatopsis* sp.	BGC0001503	amycolamycin A, B	532	45	63	2e-125
LobT (ALA09373)	*Micromonospora* sp. *RL09-050-HVF-A*	BGC0001303	lobosamide A, B, C	577	45	63	4e-124
Strop_2765 (ABP55207)	*Salinispora tropica CNB-440*	BGC0000142	salinilactam	584	44	61	7e-124
Frig2 (QDG00808)	*Streptomyces griseus*	BGC0002028	frigocyclinone	539	47	61	6e-124
Med-ORF25 (BAC79020)	*Streptomyces* sp. *AM-7161*	BGC0000245	medermycin	525	47	61	1e-122
HerN (AKD43759)	*Streptomyces* sp. *SCSIO 03032*	BGC0001349	heronamide A, B, C, D, E, F	520	44	62	2e-120
Orf14 (QBL56188)	*Streptomyces* sp.	BGC0002376	bombyxamycin A, B	531	41	60	2e-119
MdpR3 (ABY66009)	*Actinomadura madurae*	BGC0001008	maduropeptin	525	42	61	4e-119

SSGG_00659 (EFE73293)	*Streptomyces filamentosus NRRL 15998*	BGC0000431	stenothricin	514	47	63	4e-112
BN6_54810 (CCH32740)	*Saccharothrix espanaensis DSM 44229*	BGC0002070	pentangumycin, SEK90	499	48	62	8e-111
FlvT1 (BAV56014)	*Actinomadura fulva subsp. indica*	BGC0001597	fluvirucin B2	506	42	60	2e-108
FZ103_09785 (MUL41461)	*Streptomonospora* sp. *PA3*	BGC0002045	persiamycin A	526	46	62	7e-107
HitT1 (BAR73016)	*Streptomyces scabrisporus*	BGC0001194	hitachimycin	538	37	54	1e-91

TABLE S6 Conserved amino acid motifs of the major facilitator superfamily (MFS) transporters (Paulsen et al. 1996) found in the CpkF sequence. The consensus sequences of the motifs (upper lines) are shown as follows: x – any amino acid, capital letter – conservative amino acid, lowercase letter – amino acid which can be replaced with a similar one. The amino acids of CpkF (lower lines) corresponding to the motifs are shown in bold. TMS – transmembrane segment.

Motif (CpkF aa span)	Sequence match	Description
**D1** (33-43)	**lD**x**Tv**x**nvAlP**<br>A**D**LG**V**LW**LA**T**P**	In TMS1, typical for 14TMS members of MFS
**A** (75-88)	**G**x**LaDr**x**Grk**xxx**l**<br>**G**T**LGDR**I**GRR**RLL**I**	In inside loop between TMS2 and TMS3; typical for all MFS members
**B** (110-122)	**l**xxx**R**xx**qG**x**gaa**<br>**L**IVA**R**ALL**G**V**AGA**	In TMS4; typical for all MFS members
**C** (156-167)	**g**xxx**GP**xx**GG**x**l**<br>**G**IAI**GP**VI**GG**V**L**	In TMS5; typical for drug–ion antiporters
**H** (173-184)	**W**x**w**x**FlINvPig**<br>**W**GSV**FLM**G**VPV**M	In TMS6, typical for 14TMS members of MFS
**E** (207-213)	**D**xx**G**xx**L**<br>**D**LA**S**VV**L**	In TMS7, typical for 14TMS members of MFS
**F** (411-422)	**lg**xxx**G**x**av**x**gl**<br>**LG**LAF**G**I**AV**T**G**S	In TMS13, typical for 14TMS members of MFS

TABLE S7 *Streptomyces coelicolor* A3(2) proteins homologous to CpkF identified by BLAST search (http://strepdb.streptomyces.org.uk/).

SCO number	Length (aa)	% of identical aa	% of similar aa	E-value
SCO1567	510	45	62	8e-132
SCO5516	521	43	59	3e-130
SCO1751	534	40	56	1e-107
SCO6822	521	34	52	9e-88
SCO5957	533	32	49	2e-72
SCO5076	533	35	50	9e-71
SCO1190	533	30	48	5e-64

SCO4024	507	30	47	1e-60

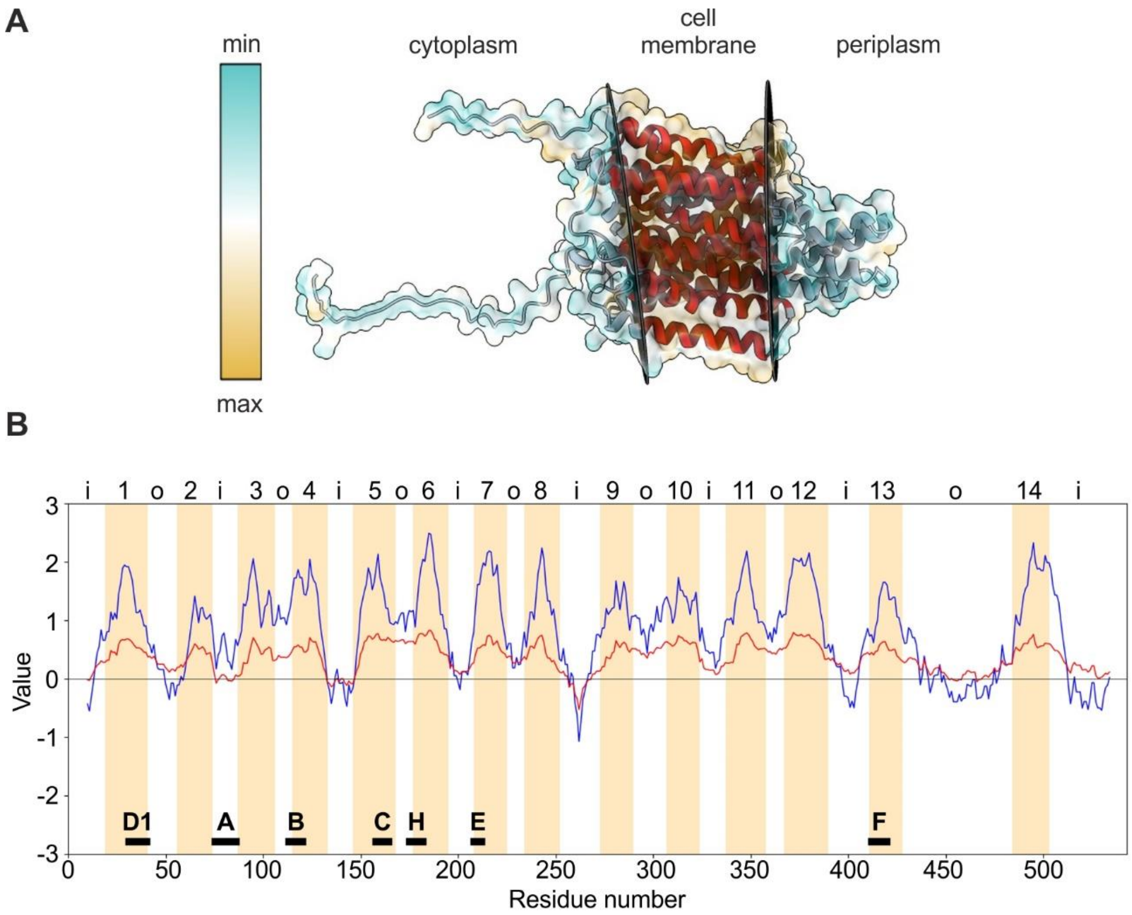

FIGURE S1. Predicted structural features of CpkF protein. (**A**) Model of CpkF structure predicted by AlphaFold (Jumper et al. 2021; Varadi et al. 2024). Protein surface hydrophobicity is displayed in cyan(min)/yellow(max) coloring. Transmembrane helices are depicted in red and are limited with the dark planes that indicate a cell membrane. Visualisation was made with UCSF ChimeraX (Meng *et al.*, 2023). (**B**) Topology of the transmembrane segments. Hydropathy (blue line) and amphipathicity (red line) plots were obtained with the WHAT program (Zhai & Saier, 2001). The orange bars mark transmembrane segments as predicted by HMMTOP (Tusnády and Simon, 2001). TMS numbers, as well as inside (i) and outside (o) loop locations, are given on top of the graph. The black bars mark the locations of the sequence motifs described in Table S4.

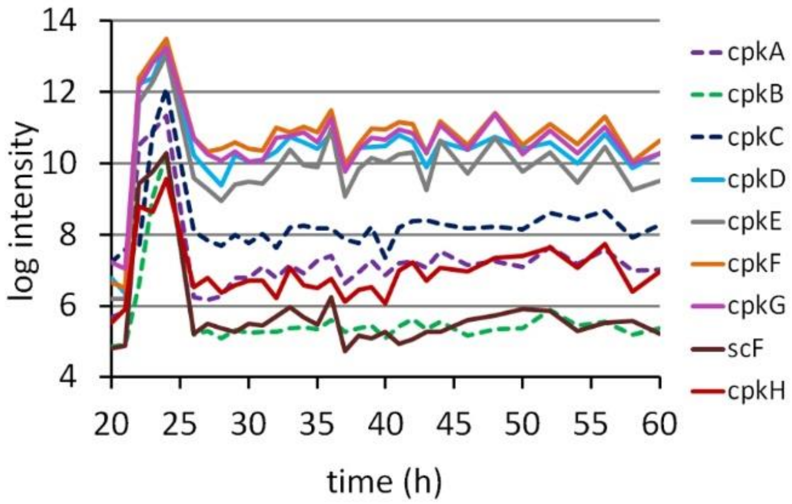

FIGURE S2. Expression profile of selected *cpk* genes. The plot is based on transcriptomic microarray data from (Nieselt et al. 2010).

Supplementary References

Bednarz, B. *et al.* (2021) 'Coelimycin Synthesis Activatory Proteins Are Key Regulators of Specialized Metabolism and Precursor Flux in *Streptomyces coelicolor* A3(2)', *Frontiers in Microbiology*, 12. Available at: https://doi.org/10.3389/FMICB.2021.616050.

Brown MH, Skurray RA (2001) Staphylococcal multidrug efflux protein QacA. J Mol Microbiol Biotechnol 3:163–170 https://www.caister.com/backlist/jmmb/v/v3/v3n2/05.pdf

Cho YH, Kim EJ, Chung HJ, Choi JH, Chater KF, Ahn BE, Shin JH, Roe JH (2003) The pqrAB Operon Is Responsible for Paraquat Resistance in *Streptomyces coelicolor*. J Bacteriol 185:6756. https://doi.org/10.1128/JB.185.23.6756-6763.2003

Craney, A. *et al.* (2007) 'A synthetic luxCDABE gene cluster optimized for expression in high-GC bacteria', *Nucleic Acids Research*, 35(6). Available at: https://doi.org/10.1093/nar/gkm086.

Gust, B. *et al.* (2004) 'λ Red-Mediated Genetic Manipulation of Antibiotic-Producing *Streptomyces*', *Advances in Applied Microbiology*, 54, pp. 107–128. Available at: https://doi.org/10.1016/S0065-2164(04)54004-2.

Hong, H.J. *et al.* (2005) 'The role of the novel Fem protein VanK in vancomycin resistance in *Streptomyces coelicolor'*, *The Journal of biological chemistry*, 280(13), pp. 13055–13061. Available at: https://doi.org/10.1074/JBC.M413801200.

Jumper, J. *et al.* (2021) 'Highly accurate protein structure prediction with AlphaFold', *Nature*, 596(7873), pp. 583–589. Available at: https://doi.org/10.1038/s41586-021-03819-2.

Kieser, T. *et al.* (2000) *Practical Streptomyces Genetics*. Norwich, UK: The John Innes Foundation.

Lee LF, Chen YJ, Kirby R, Chen C, Chen CW (2007) A multidrug efflux system is involved in colony growth in *Streptomyces lividans*. Microbiology (Reading) 153:924–934. https://doi.org/10.1099/MIC.0.2006/000018-0

Lee LF, Huang YJ, Chen CW (2003) Repressed multidrug resistance genes in *Streptomyces lividans*. Arch Microbiol 180:176–184. https://doi.org/10.1007/s00203-003-0574-z

Medema et al. (2015) Minimum Information about a Biosynthetic Gene cluster. Nat Chem Biol 11:625–631. https://doi.org/10.1038/NCHEMBIO.1890

Meng, E.C. *et al.* (2023) 'UCSF ChimeraX: Tools for structure building and analysis', *Protein Science*, 32(11). Available at: https://doi.org/10.1002/pro.4792.Nieselt, K. *et al.* (2010) 'The dynamic architecture of the metabolic switch in *Streptomyces coelicolor*', *BMC Genomics*, 11(1). Available at: https://doi.org/10.1186/1471-2164-11-10.

Paulsen, I.T., Brown, M.H. and Skurray, R.A. (1996) 'Proton-dependent multidrug efflux systems', *Microbiological Reviews*, 60(4), pp. 575–608. Available at: https://doi.org/10.1128/MR.60.4.575-608.1996.

Pawlik, K. *et al.* (2007) 'A cryptic type I polyketide synthase (cpk) gene cluster in *Streptomyces coelicolor* A3(2)', *Archives of Microbiology*, 187(2), pp. 87–99. Available at: https://doi.org/10.1007/S00203-006-0176-7/FIGURES/3.

Pawlik, K.J. *et al.* (2021) 'GntR-like SCO3932 Protein Provides a Link between Actinomycete Integrative and Conjugative Elements and Secondary Metabolism', *International journal of molecular sciences*, 22(21). Available at: https://doi.org/10.3390/IJMS222111867.

Périchon B, Bogaerts P, Lambert T, Frangeul L, Courvalin P, Galimand M (2008) Sequence of Conjugative Plasmid pIP1206 Mediating Resistance to Aminoglycosides by 16S rRNA Methylation and to Hydrophilic Fluoroquinolones by Efflux. Antimicrob Agents Chemother 52:2581. https://doi.org/10.1128/AAC.01540-07

Rodrigues L, Ramos J, Couto I, Amaral L, Viveiros M (2011) Ethidium bromide transport across Mycobacterium smegmatis cell-wall: correlation with antibiotic resistance. BMC Microbiol 11:35. https://doi.org/10.1186/1471-2180-11-35

Saier MH, Reddy VS, Moreno-Hagelsieb G, Hendargo KJ, Zhang Y, Iddamsetty V, Lam KJK, Tian N, Russum S, Wang J, Medrano-Soto A (2021) The Transporter Classification Database (TCDB): 2021 update. Nucleic Acids Res 49:D461–D467. https://doi.org/10.1093/NAR/GKAA1004

Szafran, M.J. *et al.* (2016) 'The coordinated positive regulation of topoisomerase genes maintains topological homeostasis in *Streptomyces coelicolor*', *Journal of Bacteriology*, 198(21), pp. 3016–3028. Available at: https://doi.org/10.1128/JB.00530-16.

Tusnády, G.E. and Simon, I. (2001) ‘The HMMTOP transmembrane topology prediction server’, *Bioinformatics (Oxford, England)*, 17(9), pp. 849–850. Available at: https://doi.org/10.1093/BIOINFORMATICS/17.9.849.

Varadi, M. *et al.* (2024) ‘AlphaFold Protein Structure Database in 2024: providing structure coverage for over 214 million protein sequences’, *Nucleic Acids Research*, 52(D1), pp. D368–D375. Available at: https://doi.org/10.1093/nar/gkad1011.

Wilkinson, C.J. *et al.* (2002) ‘Increasing the efficiency of heterologous promoters in actinomycetes’, *Journal of Molecular Microbiology and Biotechnology*, 4(4), pp. 417–426.

Zhai, Y. and Saier, J. (2001) ‘A web-based program (WHAT) for the simultaneous prediction of hydropathy, amphipathicity, secondary structure and transmembrane topology for a single protein sequence.’, *Journal of Molecular Microbiology and Biotechnology*, 3(4), pp. 501–502. Available at: https://europepmc.org/article/med/11545267 (Accessed: 30 August 2022).
